# Population-based dementia prediction model using Korean public health examination data: A cohort study

**DOI:** 10.1371/journal.pone.0211957

**Published:** 2019-02-12

**Authors:** Kyung Mee Park, Ji Min Sung, Woo Jung Kim, Suk Kyoon An, Kee Namkoong, Eun Lee, Hyuk-Jae Chang

**Affiliations:** 1 Department of Psychiatry, Yonsei University College of Medicine, Seoul, South Korea; 2 Institute of Behavioral Science in Medicine, Yonsei University College of Medicine, Seoul, South Korea; 3 Division of Cardiology, Severance Cardiovascular Hospital, Yonsei University College of Medicine, Seoul, South Korea; 4 Department of Psychiatry, Myongji Hospital, Goyang, Gyeonggi, South Korea; 5 Severance Biomedical Science Institute, Yonsei University College of Medicine, Seoul, South Korea; University of Haifa, ISRAEL

## Abstract

The early identification and prevention of dementia is important for reducing its worldwide burden and increasing individuals’ quality of life. Although several dementia prediction models have been developed, there remains a need for a practical and precise model targeted to middle-aged and Asian populations. Here, we used national Korean health examination data from adults (331,126 individuals, 40–69 years of age, mean age: 52 years) from 2002–2003 to predict the incidence of dementia after 10 years. We divided the dataset into two cohorts to develop and validate of our prediction model. Cox proportional hazards models were used to construct dementia prediction models for the total group and sex-specific subgroups. Receiver operating characteristics curves, C-statistics, calibration plots, and cumulative hazards were used to validate model performance. Discriminative accuracy as measured by C-statistics was 0.81 in the total group (95% confidence interval (CI) = 0.81 to 0.82), 0.81 in the male subgroup (CI = 0.80 to 0.82), and 0.81 in the female subgroup (CI = 0.80 to 0.82). Significant risk factors for dementia in the total group were age; female sex; underweight; current hypertension; comorbid psychiatric or neurological disorder; past medical history of cardiovascular disease, diabetes mellitus, or hypertension; current smoking; and no exercise. All identified risk factors were statistically significant in the sex-specific subgroups except for low body weight and current hypertension in the female subgroup. These results suggest that public health examination data can be effectively used to predict dementia and facilitate the early identification of dementia within a middle-aged Asian population.

## Introduction

Dementia is a major public health concern within the older population. In 2012, the World Health Organization (WHO) estimated that 36 million people were living with dementia. With demographic changes and longer life expectancies, more than 115 million people are expected to have dementia by 2050. At this rate, the total cost of the disease will rise from €515 billion to €16 trillion per year [1]. At the individual level, dementia shortens life expectancy and decreases quality of life. Dementia is estimated to contribute 11.2% of years lived with disability among individuals >60 years of age compared with 9.5% for stroke and 5.0% for cardiovascular disease [2].

Research on dementia treatment, including that for Alzheimer’s dementia (AD), has made remarkable progress, but treatment efficacy is still limited [3]. Thus, the early identification of individuals at a high risk of dementia and correction of modifiable risk factors are crucial. About one-third of cases of AD, which is the most frequent type of dementia, is attributable to modifiable risk factors [4]. It is assumed that a 10–25% reduction in modifiable risk factors could prevent 3 million cases of AD, and delaying the onset of dementia by 1 year could lower the incidence of dementia by more than 9 million over the next 40 years [5]. There is also a close relationship between AD and vascular dementia (VD), which is the second most common type of dementia [6,7]. Epidemiological studies show that AD and VD share common risk factors such as hypertension, diabetes mellitus, smoking, hypercholesterolemia, and age [8–10]. Pathological studies also suggest overlap between AD and vascular pathology in older adults with dementia [11,12]. Therefore, the development of a prediction model for all-cause dementia, including both AD and VD, could have several practical benefits.

Some existing dementia prediction models use clinic-based criteria to detect mild cognitive impairment, whereas others use more comprehensively constructed algorithms including information such as demographic (e.g., age, sex, education) and health-related (e.g., comorbid diseases, laboratory data, lifestyle information) characteristics [13]. Such models can be broadly categorized into demographic-only models, cognitive-based models, health risk models, risk models using genetic information, and multi-variable models [14]. Although prediction models using genetic or neuroimaging data can be particularly informative [15,16], they are too expensive for primary prevention.

Also, most existing dementia prediction models are designed for an older population >65 years of age. Around 60–70% of aging-related dementia is usually diagnosed after the age of 65 [17], but the disease process may start much earlier. To allow early identification of high-risk individuals and modification of risk factors, dementia prediction models must be developed for a mid-life population. Furthermore, few existing prediction models target Asian populations, especially Koreans. Therefore, we sought to develop a practical and precise dementia prediction model for a Korean middle-aged population.

In South Korea, the National Health Insurance Service (NHIS) conducts a health examination for all citizens >40 years of age every 2 years. The NHIS stores all health examination data and national records of medical utilization and prescriptions for billing purposes. For research purposes, the NHIS constructs the Health Screening Cohort (NHIS-HEALS) dataset through anonymization [18]. More than 500,000 randomly sampled individuals are included in NHIS-HEALS, representing 10% of the entire Korean population. The objective of this study was to develop a dementia prediction model using health examination data from NHIS-HEALS for middle-aged Koreans.

## Materials and methods

### Study population and sample selection

We used baseline NHIS-HEALS data from the years 2002 and 2003. As our primary outcome was the incidence of all-cause dementia, we identified the following medical diagnostic codes according to the International Classification of Diseases 10^th^ edition (ICD-10): dementia in Alzheimer’s disease (F00), vascular dementia (F01), dementia in Pick’s disease (F02), unspecified dementia (F03), and Alzheimer’s disease (G30). We selected these codes as our target variable and excluded cases with codes for dementia at baseline.

A total of 473,036 individuals (259,259 males and 213,777 females) between the ages of 40 and 69 years with no history of any dementia at baseline were included. We included the following variables in our analysis: age, sex, body mass index (BMI), systolic blood pressure (SBP), diastolic blood pressure (DBP), fasting blood glucose, past medical history (i.e., cardiovascular disease, diabetes, or hypertension), current comorbid psychiatric or neurologic disorders, current smoking, and exercise. After reviewing previous studies on risk factors for dementia, these variables were chosen by expert psychiatrists (SK An, K Namkoong, and E Lee). Explanatory variables, including height, weight, SBP, and DBP, were obtained directly from the health examination. Other variables, including age, medical history, smoking, and exercise, were obtained from questionnaires administered during the health examination. BMI was calculated as weight in kilograms divided by the square of height in meters. In addition, we obtained information on comorbid psychiatric or neurologic diseases in NHIS-HEALS at baseline. We excluded individuals with a diagnosis of dementia at baseline or who died within the first 2 years of observation.

**Fig 1:**
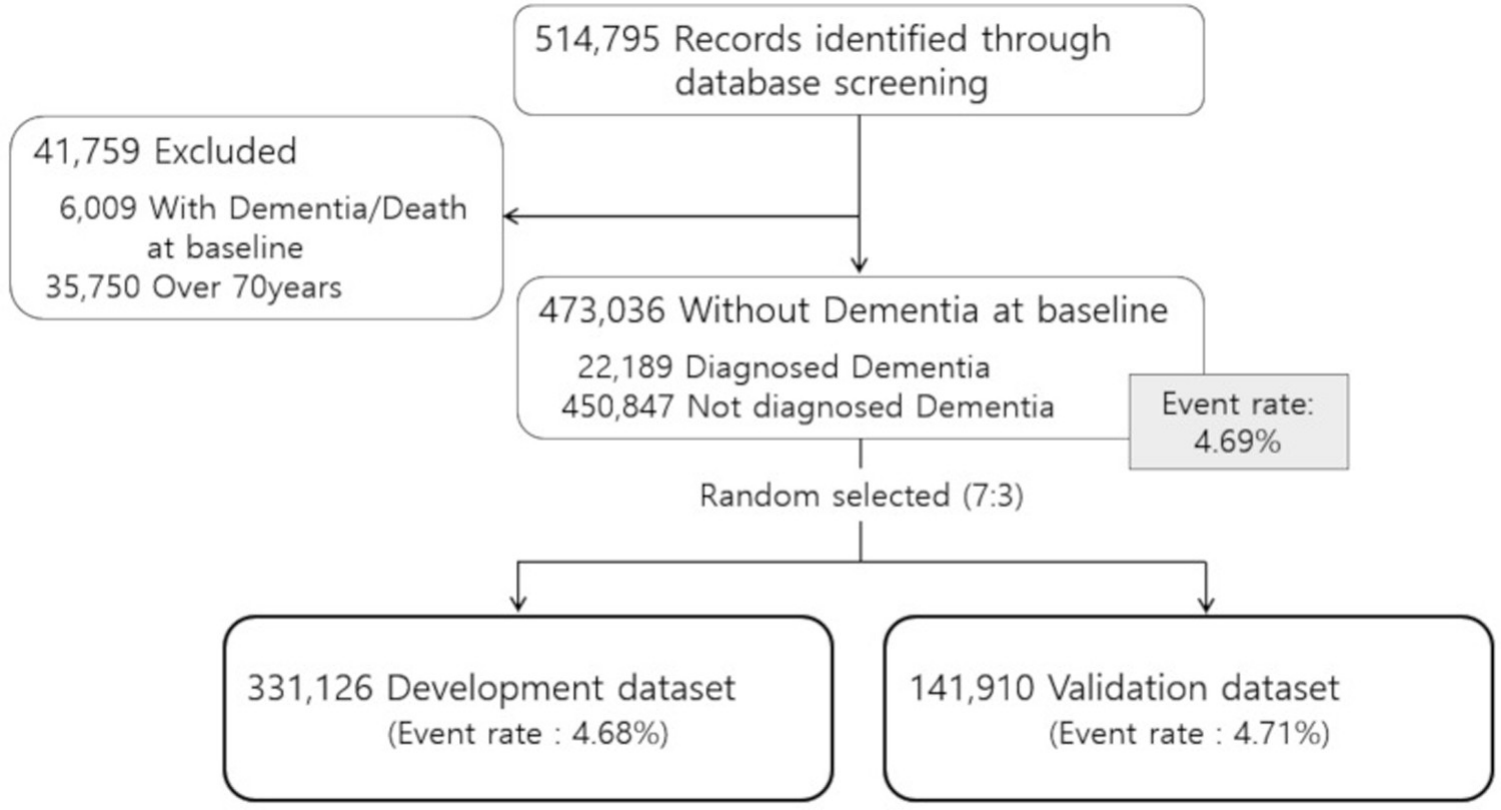
Flowchart of cohort data processing.

For construction of a dementia prediction model, we divided the baseline data into two datasets: a development (70%) and a validation (30%) dataset. The development dataset was used to fit the parameters of classifiers to establish a prediction model. Validation datasets were used to assess the accuracy of the final model. We used simple random sampling to divide the two datasets regardless of the occurrence of dementia (Fig 1). For missing data, the mean imputation was used for the whole study sample at every time they gathered by using SAS MI (multiple imputation) procedure [19]. This study was approved by the Institutional Review Board of the National Cancer Center, Korea (IRB No. 4-2016-0383). The need for participant consent was waived by the ethics committee because this study involved routinely collected medical data that were anonymously managed at all stages, including data cleaning and statistical analysis.

### Statistical analysis and prediction model development

The primary outcome of this study was the incidence of all-cause dementia including dementia in Alzheimer’s disease (F00), vascular dementia (F01), dementia in Pick’s disease (F02), unspecified dementia (F03), or Alzheimer’s disease (G30). We defined this event as occurring when an individual was first diagnosed with dementia corresponding to an ICD-10 code. We followed individuals from the first health examination date during the baseline period (2002–2003) until December 31, 2013. The time to event was defined as the time from the first examination date to the diagnosis date. If an individual was not diagnosed with dementia during the follow-up period, the time to event was defined as the time from the first examination date to the most recent date among the last health examination date, the last hospital visit date, and the date of death. Cases of loss to follow up or death without a dementia diagnosis were considered as cases without a dementia event, and were censored.

Cox proportional hazards models were used to construct the total and sex-specific subgroup models using baseline data. The time to event was 10.24 ± 1.73 years (mean ± standard deviation) in the total group, 10.26 ± 1.78 years in the male subgroup, and 10.21 ± 1.66 years in the female subgroup in the development cohort and 10.23 ± 1.73 years in the total group, 10.26 ± 1.79 years in the male subgroup, and 10.20 ± 1.67 years in the female subgroup in the validation cohort.

Explanatory variables included age (5 years), sex (male or female), BMI (underweight: BMI < 18.5, normal: 18.5 ≤ BMI < 23, overweight: 23 ≤ BMI < 25, obese: BMI ≥ 25 hypertension (normotensive: SBP < 120mmHg and DBP < 80mmHg, prehypertensive: 120mmHg ≤ SBP < 140mmHg and 80mmHg ≤ DBP < 90mmHg, hypertensive I: 140mmHg ≤ SBP < 160 mmHg and 90 mmHg ≤ DBP < 100 mmHg, hypertensive II: SBP ≥ 160 mmHg and DBP ≥ 100 mmHg), current smoking status (currently smoking or has smoked >100 cigarettes in their life), regular exercise (>30 minutes a week), past medical history (cardiovascular disease, diabetes mellitus, or hypertension), and comorbid psychiatric or neurologic disorders. Comorbid psychiatric disorders included the following diagnoses: organic amnesic syndrome not induced by alcohol or other psychoactive substances (F04), delirium not induced by alcohol or other psychoactive substances (F05); other mental disorders due to brain damage and dysfunction or to physical disease (F06); personality and behavioral disorders due to brain disease, damage, or dysfunction (F07); unspecified organic or symptomatic mental disorders (F09); schizophrenia, schizotypal, or delusional disorders (F2x); mood (affective) disorders (F3x); mental retardation (F7x); and disorders of psychological development (F8x). Neurologic disorders included the following diagnoses: inflammatory diseases of the central nervous system (G0x), systemic atrophies primarily affecting the central nervous system (G1x), extrapyramidal and movement disorders (G2x), other degenerative diseases of the nervous system not elsewhere classified (G31), other degenerative disorders of the nervous system in diseases classified elsewhere (G32), multiple sclerosis (G35), other acute disseminated demyelination (G36), other demyelinating diseases of the central nervous system (G37), and episodic and paroxysmal disorders (G4x).

Cases with missing variables were included in analysis using the fully conditional specification (FCS) method, a multiple imputation algorithm, to compensate for the missing values. Because the FCS approach imputes data on a variable-by-variable basis by specifying an imputation model per variable, this allows tremendous flexibility in creating multivariate models.

To develop the dementia prediction model, Cox proportional hazards regression was performed to analyze the onset of dementia. The probability of dementia (P) at t years for an individual with K risk factors was estimated as follows:

$$\text{P}=1-{\text{S(t)}}^{\text{exp}(f[x,M])}\;\;\;f(x.M)=\beta_{1}(x_{1}-M_{1})+\ldots \ldots +\beta_{0}(x_{p}-M_{p}),$$

where S(t) is the survival rate at the mean value of the risk factor at 10 years, $\beta_{1}\cdots \cdots \beta_{p}$ are the regression coefficients, $x_{1}\cdots \cdots x_{p}$ represent an individual’s risk factors at baseline, and $M_{1}\cdots \cdots M_{p}$ are the mean values of the risk factors in NHIS-HEALS.

To validate the dementia prediction model, we evaluated model performance by measuring its ability to discriminate between event and non-event cases using receiver operating characteristic (ROC) curves, C-statistics, calibration plots, and cumulative hazards. ROC curves and C-statistics were used to evaluate the ability of the model to predict a binary outcome [20]. We performed discrimination analysis of the model to assess its performance. We also used bootstrap method for the calculation of area under the cover (AUC) to estimate corresponding confidence intervals. Calibration plot is a method about the estimated probabilities and meant to present how well the classifier is calibrated [21]. In this study, it was used to determine the similarity of the actual target variable to the predicted variable [22]. Hosmer-Lemeshow (H-L) tests were used for calibration, which was performed by splitting the observations into five equal-sized risk groups and comparing the observed proportions of event outcomes to the mean predicted proportions of events within each group [23]. Lastly, we calculated cumulative hazards for the five risk groups divided by their prediction probabilities. All statistical analyses were performed using SAS 9.4 (SAS Institute, North Carolina, USA) and R version 3.4 (The R Foundation for Statistical Computing, Vienna, Austria).

## Results

Table 1 shows baseline characteristics of the development cohort from NHIS-HEALS (40–69 years of age), including the total group and sex-specific subgroups. Of the total 331,126 individuals (181,500 males and 149,626 females) in the development cohort, 15,501 (6,664 males and 8,837 females) were diagnosed with dementia, with an event rate of 4.7% (3.7% for males and 6.0% for females) during 10 years of follow up (S1 Table). Baseline characteristics of the validation cohort, including the total group and sex-specific subgroups, are shown in S2 Table. All characteristics exhibited significant sex differences in both the development and validation cohorts.

**Table 1:** Baseline characteristics of the development cohort.

Variable		Total (n = 331,126)	Males (n = 181,500)	Females (n = 149,626)	p-value
Duration of follow up, years		10.37 ± 1.54	10.39 ± 1.60	10.35 ± 1.46	<0.0001
Age, years		51.41 ± 8.13	50.84 ± 8.03	52.11 ± 8.21	<0.0001
BMI, n (%)	Underweight (<18.5)	6,554 (1.98)	3,585 (1.98)	2.969 (1.98)	<0.0001
	Normal (18.5 ≤ n < 23)	115,736 (34.95)	60,069 (33.10)	55,667 (37.32)	<0.0001
	Overweight (23 ≤ n < 25)	90,804 (27.42)	51,569 (28.41)	39,235 (26.22)	<0.0001
	Obese (≥25)	118,032 (35.65)	66,277 (36.52)	51,755 (34.59)	<0.0001
Hypertension, n (%)	Normotensive (SBP < 120 and DBP < 80)	85,906 (25.94)	36,892 (20.33)	49,014 (32.76)	<0.0001
	Prehypertensive (120 ≤ SBP < 140 and 80 ≤ DBP < 90)	179,989 (54.36)	104,097 (57.35)	75,892 (50.72)	<0.0001
	Hypertensive I (140 ≤ SBP < 160 and 90 ≤ DBP < 100)	52,860 (15.96)	32,787 (18.06)	20,073 (13.42)	<0.0001
	Hypertensive II (SBP ≥ 160 and DBP ≥ 100)	12,371 (3.74)	7,724 (4.26)	4,647 (3.11)	<0.0001
Known past history, n (%)	Cardiovascular disease	21,551 (6.51)	10,516 (5.79)	11,035 (7.38)	<0.0001
	Diabetes mellitus	12,885 (3.89)	7,500 (4.13)	5,385 (3.60)	<0.0001
	Hypertension	24,386 (7.36)	11,215 (6.18)	13,171 (8.80)	<0.0001
Psychiatric disorder, n (%)		10,824 (3.27)	3,655 (2.01)	7,169 (4.79)	<0.0001
Neurological disorder, n (%)		28,686 (8.66)	9,639 (5.31)	19.047 (12.73)	<0.001
Current smoking, n (%)		81,727 (24.68)	77,295 (42.59)	4,426 (2.96)	<0.001
Regular exercise, n (%)		144,431 (43.62)	93,152 (51.32)	51,279 (34.27)	<0.001
Dementia event, n (%)		15,501 (4.7)	6,664 (3.7)	8,837 (6.0)	<0.001

P-values indicate differences between male and female subgroups. BMI, body mass index; SBP, systolic blood pressure; DBP, diastolic blood pressure.

**Fig 2:**
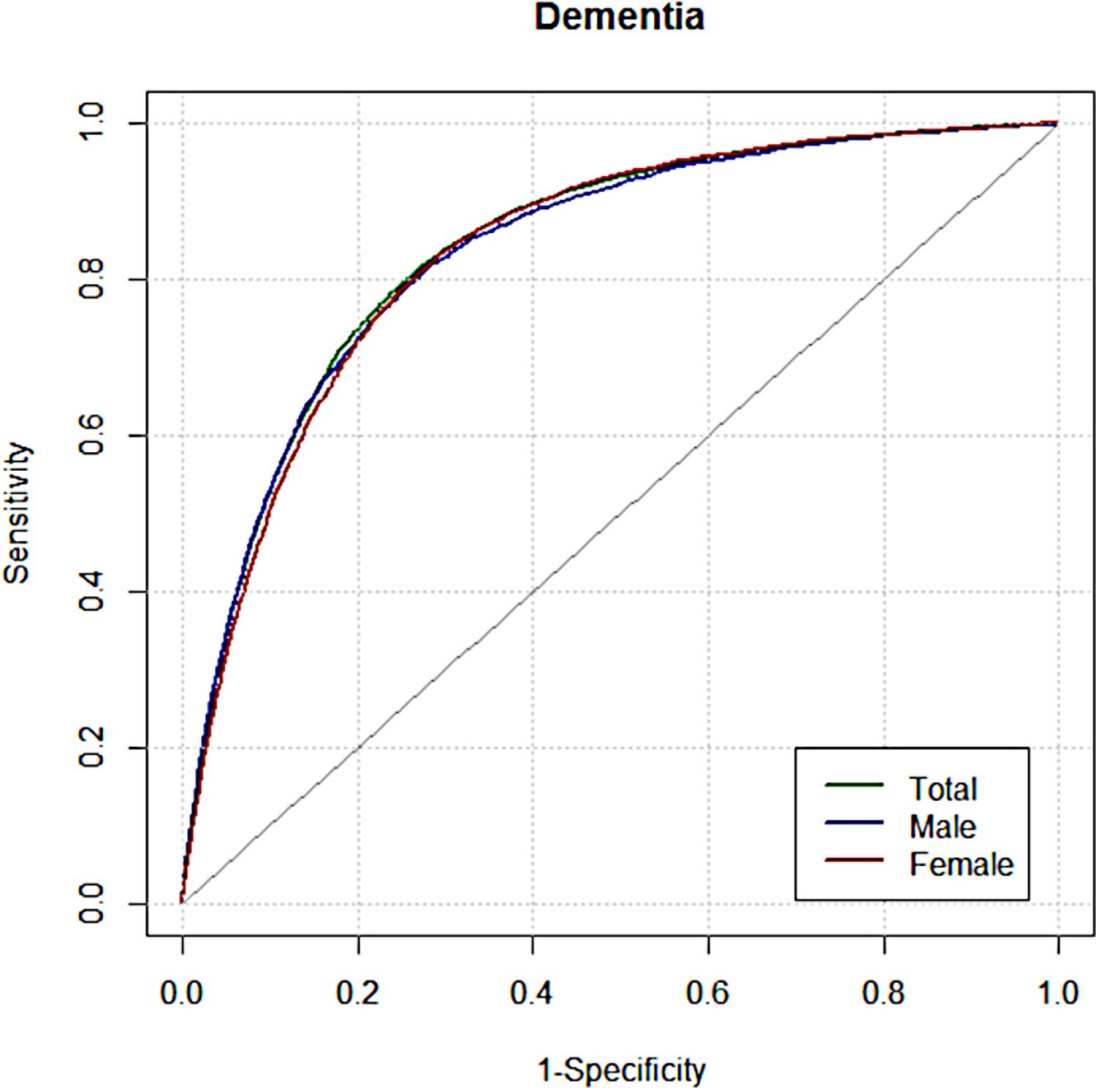
ROC curves for dementia prediction model.

We calculated hazard ratios for dementia for the total group (Table 2) and sex-specific subgroups (S3 Table) to construct prediction models using Cox proportional hazards regression analysis. ROC curves for the total group and sex-specific subgroups are presented in Fig 2. Discriminative accuracy measured by C-statistics was 0.81 for the total group (95% confidence interval (CI) = 0.81 to 0.82), 0.81 for the male subgroup (CI = 0.80 to 0.82), and 0.81 in the female subgroup (CI = 0.80 to 0.82). Further discrimination analyses can be seen in S4 Table. Concordance measured by AUC of 1000 times of bootstrap-estimated value was 0.81 for the total group (CI = 0.81 to 0.81), 0.81 for the male subgroup (CI = 0.81 to 0.81), 0.81 for the female subgroup (CI = 0.81 to 0.81). H-L tests for the calibration plots were performed for the total group and sex-specific subgroups (S1 and S2 Figs). We also calculated cumulative hazard ratios for five risk groups (S3 Fig).

**Table 2:** Hazard ratios for baseline variables in the development cohort.

		Total (n = 331,126)		
Variable		Hazard Ratio	95% Cl	p-value
Age (5 years)		1.99	1.97–2.02	<0.0001
Sex (ref = males)		1.32	1.27–1.37	<0.0001
BMI (ref = normal)	Underweight (<18.5)	1.12	1.01–1.24	0.0285
	Overweight (23 ≤ n < 25)	0.93	0.90–0.97	0.0009
	Obese (≥25)	0.92	0.89–0.96	<0.0001
Hypertension (ref = normal)	Prehypertensive (120 ≤ SBP < 140 and 80 ≤ DBP < 90)	1.03	1.02–1.11	0.0084
	Hypertensive I (140 ≤ SBP < 160 and 90 ≤ DBP < 100)	1.11	1.06–1.17	<0.0001
	Hypertensive II (SBP ≥ 160 and DBP ≥ 100)	1.18	1.08–1.27	<0.0001
Known past history	Cardiovascular disease	1.44	1.38–1.51	<0.0001
	Diabetes mellitus	1.53	1.45–1.62	<0.0001
	Hypertension	1.12	1.07–1.17	<0.0001
Psychiatric disorder		1.59	1.50–1.69	<0.0001
Neurological disorder		1.40	1.34–1.46	<0.0001
Current smoking		1.16	1.11–1.21	<0.0001
No exercise		1.18	1.14–1.22	<0.0001

BMI, body mass index; SBP, systolic blood pressure; DBP, diastolic blood pressure; ref, reference; CI, confidence interval.

**Fig 3:**
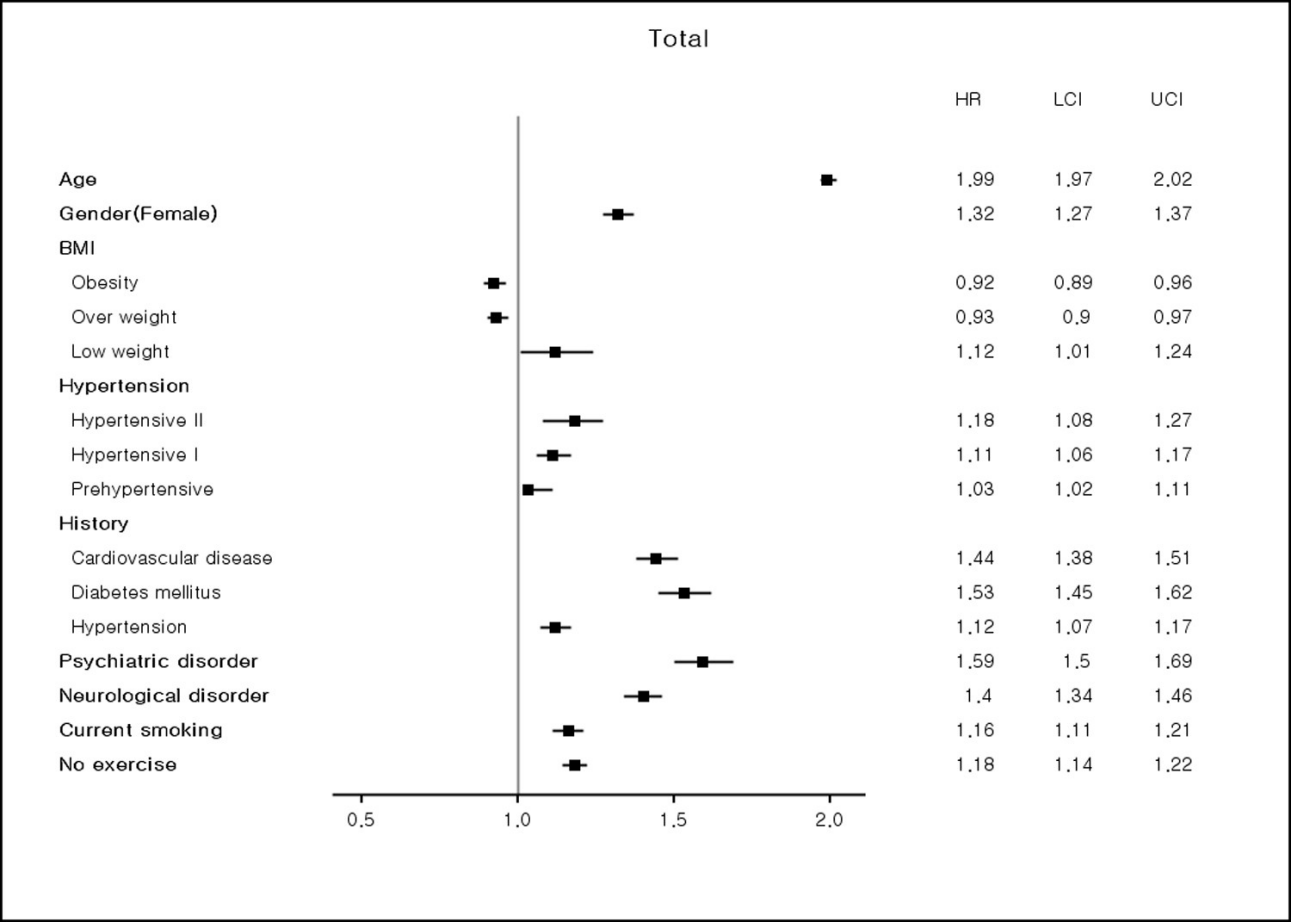
Hazard ratios for risk factors in the total group.

Our prediction model identified the following risk factors for dementia in the total group: age; female sex; underweight (BMI < 18.5); current hypertension (SBP ≥ 120 mmHg and DBP ≥ 80 mmHg); any comorbid psychiatric or neurological disorder; any past medical history of cardiovascular disease, diabetes mellitus, or hypertension; current smoking; and no exercise (Fig 3). In the male subgroup, all identified risk factors in the total group analysis were statistically significant (S4 Fig). Although the hazard ratios in the female subgroup were similar to those in the total and male subgroups, underweight and current hypertension did not reach statistical significance (S4 Fig).

## Discussion

We used national health examination data from Korean individuals aged 40–69 years to predict the development of dementia over 10 years. Using a development cohort, we successfully constructed a dementia prediction model with a good discrimination index comparable to that in some previous cohort studies [24–26]. In addition, with a validation cohort, we validated our prediction model using ROC curves, C-statistics, calibration plots, and cumulative hazards, the results of which demonstrate good performance of the model.

The discriminative accuracy of our model was constructed from health examination data obtained by simple laboratory tests, self-report questionnaires, and brief interviews. This good discriminative accuracy of our model is comparable to or better than that of other multi-variable or demographic data-only dementia prediction models that have discriminative accuracies ranging from 0.49 to 0.89 [14]. Therefore, in terms of balancing discriminative accuracy and efficiency of data collection [27], the present model may be desirable for the early detection of high-risk individuals. The finding of this study can be used for patient education about future dementia risk, especially middle-aged individuals who have several risk factors which found related to dementia occurrence.

Our cohort was consisted of middle-aged individuals from South Korea, who were expected to undergo a health examination every 2 years. Thus, this cohort represents the characteristics of the total population of middle-aged Koreans. Population-based studies have several advantages [28]. In addition to providing prevalence rates of relevant variables in the reference population, they allow comparisons with future cross-sectional studies. They are also ideal for carrying out unbiased evaluations of relationships between variables. Therefore, our model allows the expansion and application of follow-up studies of populations in Korea or other countries.

To our knowledge, we are the first to develop a dementia prediction model for Koreans. Also, this is the first model developed using simple health examination data from Asians, although two other dementia prediction models already exist for Asians [29,30]. One model using data from Japanese adults aged 60–79 years reports risk factors similar to those in our study [30]. However, the primary goal of this Japanese study was to examine the effect of the APOE allele on dementia onset, which had a very different focus than that of our study. The other model, which was modified from the one developed for non-Asian people, was found to work well for Chinese older adults [29]. However, there is still a lack of studies examining whether dementia prediction models for certain ethnic groups are applicable to other ethnic groups. In addition, no studies have examined the effect of ethnicity on dementia risk factors.

Another strength of our model is that it targets a middle-aged population. To allow the early identification and modification of risk factors, it would be optimal to develop a dementia prediction model for middle-aged people [31]. There are a few comparable multi-variable prediction models targeting middle-aged populations [24,26,31]. All the models were constructed using data from non-Asian people and had discriminative accuracies between 0.7 and 0.8. One was a model constructed using level of education, occupational attainment, and APOE status [31]. The other two were CAIDE risk score models constructed using simple demographic data and health-related features [24,26]. One CAIDE risk score model identifies age, low education, hypertension, hypercholesterolemia, and obesity as predictive of AD [26]. Although some of our risk factors, such as age and hypertension, are similar to those in CAIDE risk score models for middle-aged cohorts, the predictive value of BMI differs among models [24,26]. In the present study, a BMI < 18.5 was a risk factor, whereas in the original CAIDE study, a BMI > 30 was predictive of dementia [26]. Furthermore, in the model that predicted dementia using CAIDE risk scores in a United States middle-aged cohort, obesity was not a pronounced risk factor [24]. Among dementia prediction studies among older adults (i.e., >65 years of age), some report obesity as a risk factor [32,33], whereas others report a low BMI as a risk factor [34]. According to a 2016 WHO report [35], average BMI differs between Asia and the United States or Europe. In the United States, United Kingdom, and Australia, the average BMI for adults >18 years of age is 28.9 (28.5–29.3), 27.1 (26.6–27.6), and 27.1 (26.8–27.5), respectively, whereas the average BMI for Korean adults is 23.8 (23.5–24.0). Moreover, the Asia-Pacific classification of BMI has lower cutoffs for overweight and obese categories compared with the WHO classification [36]. Thus, the low obesity rate in our cohort may have contributed to no association between obesity and dementia onset. Furthermore, meta-analyses suggest a possibility of a U-shaped relationship between BMI and dementia in middle-aged populations [37,38]. A recent prospective study conducted in a French cohort shows that rapid weight loss in people >65 years of age can contribute to the development of dementia as a preclinical change or a result of malnutrition [39]. BMI is considered a modifiable risk factor along with smoking and alcohol drinking. However, further studies are needed to determine the optimal modification of BMI and to compare the association of BMI with dementia between different countries. The effects of risk factor modification on the development of dementia should also be investigated prospectively.

This study has some limitations. First, we did not include measurements of cognitive function. Although we intended to predict dementia without any measurement of cognitive function, gathering this information could have allowed us to compare the performance of our model with that of previous models and to further validate its predictability. Second, we targeted all-cause dementia, not specifically AD or VD. Thus, our prediction model using health examination data is not appropriate for identifying or predicting risk factors that could contribute to the occurrence of specific dementias. However, AD and VD share several risk factors, and most cases of dementia involve brain damage with a neurodegenerative or vascular insult [40]. Therefore, our model could be used for predicting overall dementia without specifying its type. Third, the follow-up period was only 10 years, which is relatively short considering that our targeted population was middle-aged people. We will continue to follow up this population and conduct further studies to overcome this limitation.

## Conclusions

Our results suggest that the dementia prediction model can be constructed using information from simple, routine health examinations of middle-aged people and can be used to detect individuals with a high risk of developing dementia and administer early interventions via lifestyle modifications.

## Supporting information

S1 Table Dementia event rate in the development and validation cohorts (7:3). (DOCX)

S2 Table Baseline characteristics of the validation cohort. (DOCX)

S3 Table Hazard ratios for baseline variables in the development cohort by sex. (DOCX)

S4 Table Model performance of dementia prediction. (DOCX)

S1 Fig Calibration plot for the total group. (TIF)

S2 Fig Calibration plots for sex-specific subgroups. (TIF)

S3 Fig Cumulative hazards for risk groups. (TIF)

S4 Fig Hazard ratios for risk factors in sex-specific subgroups. (TIF)
