# Correction: Population-based dementia prediction model using Korean public health examination data: A cohort study

**DOI:** 10.1371/journal.pone.0225553

**Published:** 2019-11-14

**Authors:** Kyung Mee Park, Ji Min Sung, Woo Jung Kim, Suk Kyoon An, Kee Namkoong, Eun Lee, Hyuk-Jae Chang

The following information is missing from the Funding statement: This study was supported by the Social Science Korea (SSK) Project through the NRF funded by the Ministry of Education, Republic of Korea (Grant number: 2017S1A3A2067165 to Y. Youm).
