# Supplementary material for: Population-based dementia prediction model using Korean public health examination data: A cohort study
Source: PLoS One. 2019 Feb 12;14(2):e0211957. doi: 10.1371/journal.pone.0211957 (PMC6372230; doi:10.1371/journal.pone.0211957)
Supplement: S1 Table — (DOCX) [file pone.0211957.s001.docx]

**S1 Table. Dementia event rate in the development and validation cohorts (7:3)**

|  | | **Total** | **Male** | **Female** |
| --- | --- | --- | --- | --- |
| Development cohort | Individuals, n | 331,126 | 181,500 | 149,626 |
|  | Dementia events, n (%) | 15,501 (4.68%) | 6,664 (3.67%) | 8,837 (5.91%) |
| Validation  cohort | Individuals, n | 141,910 | 77,759 | 64,151 |
|  | Dementia events, n (%) | 6,688 (4.71%) | 2,869 (3.69%) | 3,819 (5.95%) |
