# Supplementary material for: Population-based dementia prediction model using Korean public health examination data: A cohort study
Source: PLoS One. 2019 Feb 12;14(2):e0211957. doi: 10.1371/journal.pone.0211957 (PMC6372230; doi:10.1371/journal.pone.0211957)
Supplement: S2 Table — (DOCX) [file pone.0211957.s002.docx]

**S2 Table. Baseline characteristics of the validation cohort**

| **Variable** | | **Total (n = 141,910)** | **Male (n = 77,759)** | **Female (n = 64,151)** | **p-value** |
| --- | --- | --- | --- | --- | --- |
| Duration of follow-up, years | | 10.37 ± 1.55 | 10.38 ± 1.63 | 10.36 ± 1.44 | <0.001 |
| Age, years | | 51.39 ± 8.12 | 50.74 ± 7.96 | 51.90 ± 8.13 | <0.001 |
| BMI, n (%) | Underweight (<18.5) | 2,801 (1.97) | 1,502 (1.93) | 1,299 (2.02) | <0.001 |
|  | Normal (18.5 ≤ n < 23) | 49,349 (34.77) | 25,531 (32.83) | 23,818 (37.13) | <0.001 |
|  | Overweight (23 ≤ n < 25) | 39,400 (27.76) | 22,496 (28.93) | 16,904 (26.35) | <0.001 |
|  | Obese (≥25) | 50,360 (35.49) | 28,230 (36.30) | 22,130 (34.50) | <0.001 |
| Hypertension, n (%) | Normotensive  (SBP < 120 and DBP < 80) | 36,616 (25.80) | 15,52 (20.05) | 21,024 (32.77) | <0.001 |
|  | Prehypertensive (120 ≤ SBP < 140 and 80 ≤ DBP < 140) | 77,276 (54.45) | 44,757 (57.56) | 32,519 (50.69) | <0.001 |
|  | Hypertensive I (140 ≤ SBP < 160 and 90 ≤ DBP < 100) | 22,768 (16.04) | 14,100 (18.13) | 8,668 (13.51) | <0.001 |
|  | Hypertensive II (SBP ≥ 160 and DBP ≥ 100) | 5,250 (3.70) | 3,310 (4.26) | 1,940 (3.02) | <0.001 |
| Known past history, n (%) | Cardiovascular disease | 9,103 (6.41) | 4,541 (5.84) | 4,562 (7.11) | <0.001 |
|  | Diabetes mellitus | 5,500 (3.88) | 3,159 (4.06) | 2,341 (3.65) | <0.001 |
|  | Hypertension | 10,377 (7.31) | 4,798 (2.08) | 5,579 (8.70) | <0.001 |
| Psychiatric disorder, n (%) | | 4,640 (3.27) | 1,616 (2.01) | 3,024 (4.71) | <0.001 |
| Neurological disorder, n (%) | | 12,312 (8.68) | 4,158 (5.35) | 8,154 (12.71) | <0.001 |
| Current smoker, n (%) | | 35,155 (24.77) | 33,145 (42.63) | 2,010 (3.13) | <0.001 |
| Regular exercise, n (%) | | 61,882 (43.61) | 39,800 (51.18) | 22,082 (34,42) | <0.001 |
| Dementia events, n | | 6,688 (4.71) | 2,869 (3.69) | 3,819 (5.95) | <0.001 |

P-values indicate differences between male and female subgroups; BMI, body mass index; SBP, systolic blood pressure; DBP, diastolic blood pressure.
