# Supplementary material for: Population-based dementia prediction model using Korean public health examination data: A cohort study
Source: PLoS One. 2019 Feb 12;14(2):e0211957. doi: 10.1371/journal.pone.0211957 (PMC6372230; doi:10.1371/journal.pone.0211957)
Supplement: S3 Table — (DOCX) [file pone.0211957.s003.docx]

**S3 Table. Hazard ratios for baseline variables in the development cohort by sex**

|  | | **Male (n = 181,500)** | | | **Female  (n = 149,626)** | | |
| --- | --- | --- | --- | --- | --- | --- | --- |
| **Variable** | | **Hazard ratio** | **95% CI** | **p-value** | **Hazard ratio** | **95% CI** | **p-value** |
| Age (5 years) | | 1.99 | 1.95–2.02 | <0.0001 | 2.00 | 1.96–2.03 | <0.0001 |
| BMI  (ref = normal) | Underweight (<18.5) | 1.19 | 1.04–1.37 | 0.0142 | 1.05 | 0.91–1.22 | 0.4951 |
|  | Overweight (23 ≤ n < 25) | 0.93 | 0.88–0.99 | 0.0275 | 0.93 | 0.88–0.98 | 0.0106 |
|  | Obese (≥25) | 0.91 | 0.86–0.97 | 0.0031 | 0.93 | 0.89–0.98 | 0.0063 |
| Hypertension (ref = normal) | Prehypertensive  (120 ≤ SBP < 140 and 80 ≤ DBP < 90) | 1.10 | 1.03–1.18 | 0.0078 | 1.04 | 0.99–1.10 | 0.1215 |
|  | Hypertensive I  (140 ≤ SBP < 160 and 90 ≤ DBP < 100) | 1.27 | 1.17–1.38 | <0.0001 | 1.01 | 0.94–1.08 | 0.7572 |
|  | Hypertensive II  (SBP ≥ 160 and DBP ≥ 100) | 1.36 | 1.21–1.52 | <0.0001 | 1.05 | 0.94–1.17 | 0.3639 |
| Known past history | Cardiovascular disease | 1.58 | 1.47–1.69 | <0.0001 | 1.35 | 1.28–1.44 | <0.0001 |
|  | Diabetes mellitus | 1.52 | 1.37–1.65 | <0.0001 | 1.54 | 1.43–1.66 | <0.0001 |
|  | Hypertension | 1.10 | 1.02–1.18 | 0.013 | 1.14 | 1.08–1.21 | <0.0001 |
| Psychiatric disorder | | 1.71 | 1.53–1.92 | <0.0001 | 1.55 | 1.44–1.67 | <0.0001 |
| Neurological disorder | | 1.52 | 1.41–1.64 | <0.0001 | 1.35 | 1.28–1.43 | <0.0001 |
| Current smoking | | 1.16 | 1.10–1.22 | <0.0001 | 1.19 | 1.07–1.33 | 0.0014 |
| No exercise | | 1.22 | 1.16–1.28 | <0.0001 | 1.14 | 1.09–1.17 | <0.0001 |

BMI, body mass index; SBP, systolic blood pressure; DBP, diastolic blood pressure, ref, reference; CI, confidence interval.
