# Supplementary material for: Population-based dementia prediction model using Korean public health examination data: A cohort study
Source: PLoS One. 2019 Feb 12;14(2):e0211957. doi: 10.1371/journal.pone.0211957 (PMC6372230; doi:10.1371/journal.pone.0211957)
Supplement: S4 Table — (DOCX) [file pone.0211957.s004.docx]

**S4 Table. Model performance of dementia prediction**

|  | **Total**  **(n = 331,126)** | **Male**  **(n = 181,500)** | **Female**  **(n = 149,626)** |
| --- | --- | --- | --- |
| Concordance (performance, 95% CI) | 0.81 (0.81 to 0.82) | 0.81 (0.80 to 0.82) | 0.81 (0.80 to 0.82) |
| Sensitivity (%, 95% CI) | 75.61 (74.58 to 76.64) | 77.31 (75.78 to 78.84) | 78.11 (76.80 to 79.42) |
| Specificity (%, 95% CI) | 73.72 (73.49 to 73.96) | 70.76 (70.43 to 71.09) | 71.48 (71.12 to 71.84) |
| Accuracy (%, 95% CI) | 73.81 (73.58 to 74.04) | 71.00 (70.68 to 71.32) | 71.87 (71.53 to 72.22) |
| Positive predictive value  (%, 95% CI) | 12.46 (12.14 to 12.78) | 9.20 (8.83 to 9.56) | 14.77 (14.29 to 15.26) |
| Negative predictive value  (%, 95% CI) | 98.39 (98.31 to 98.47) | 98.79 (98.69 to 98.88) | 98.10 (97.97 to 98.23) |

CI, confidence interval.
