# Supplementary figures and images for: Population-based dementia prediction model using Korean public health examination data: A cohort study
Source: PLoS One. 2019 Feb 12;14(2):e0211957. doi: 10.1371/journal.pone.0211957 (PMC6372230; doi:10.1371/journal.pone.0211957)

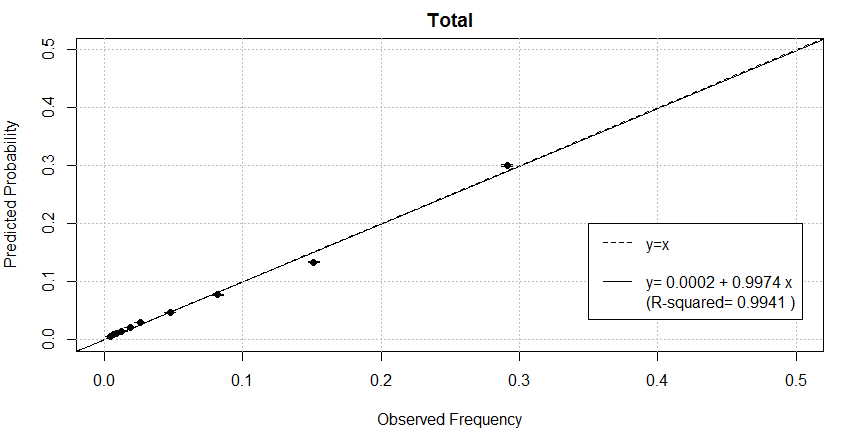

Supplement: S1 Fig — (TIF) [file pone.0211957.s005.tif]

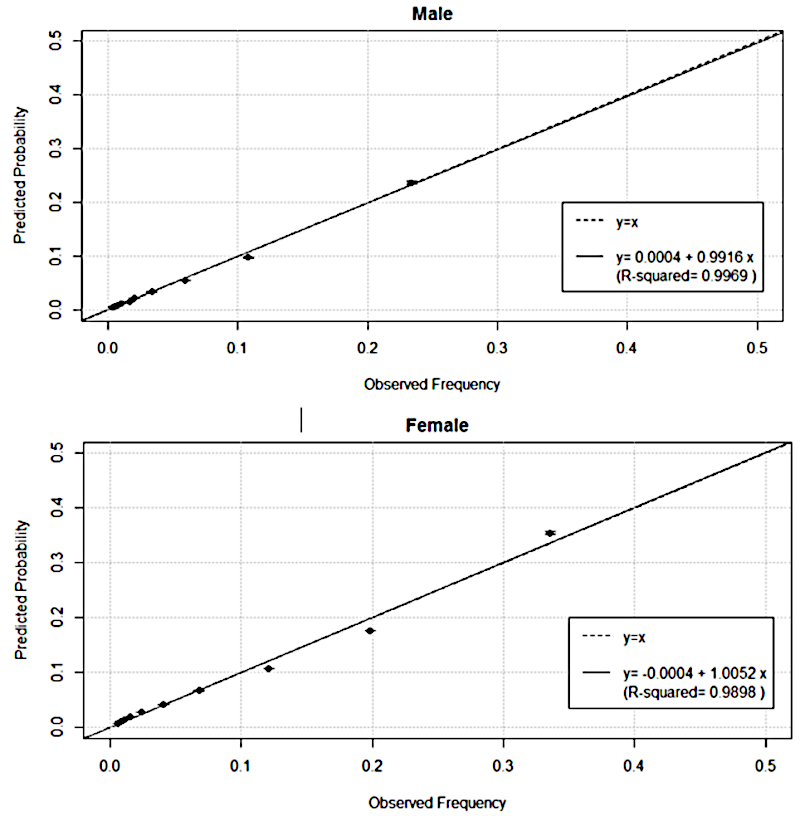

Supplement: S2 Fig — (TIF) [file pone.0211957.s006.tif]

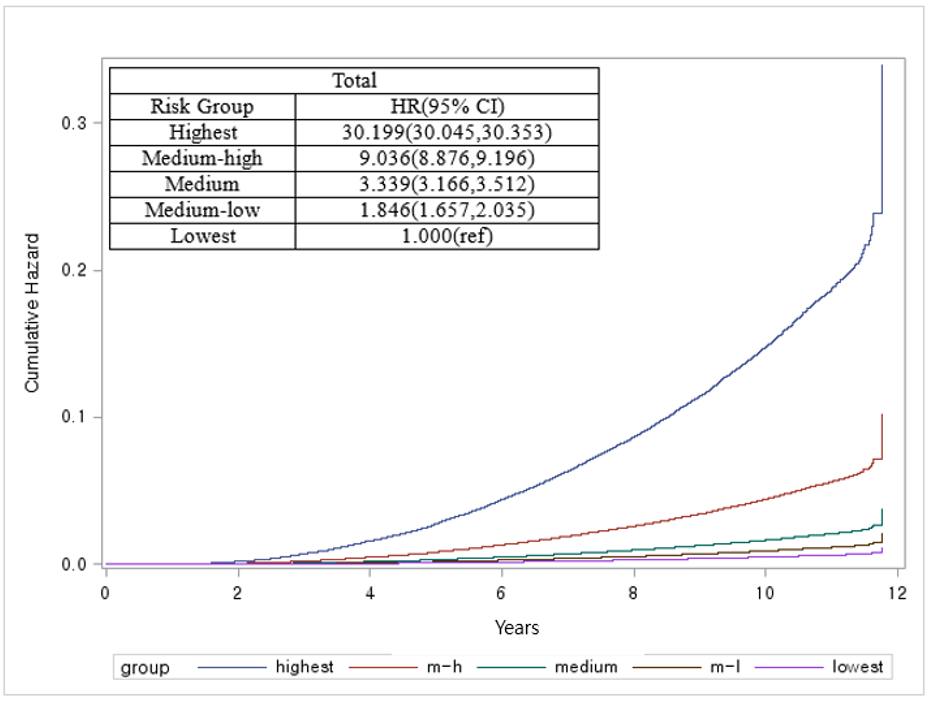

Supplement: S3 Fig — (TIF) [file pone.0211957.s007.tif]

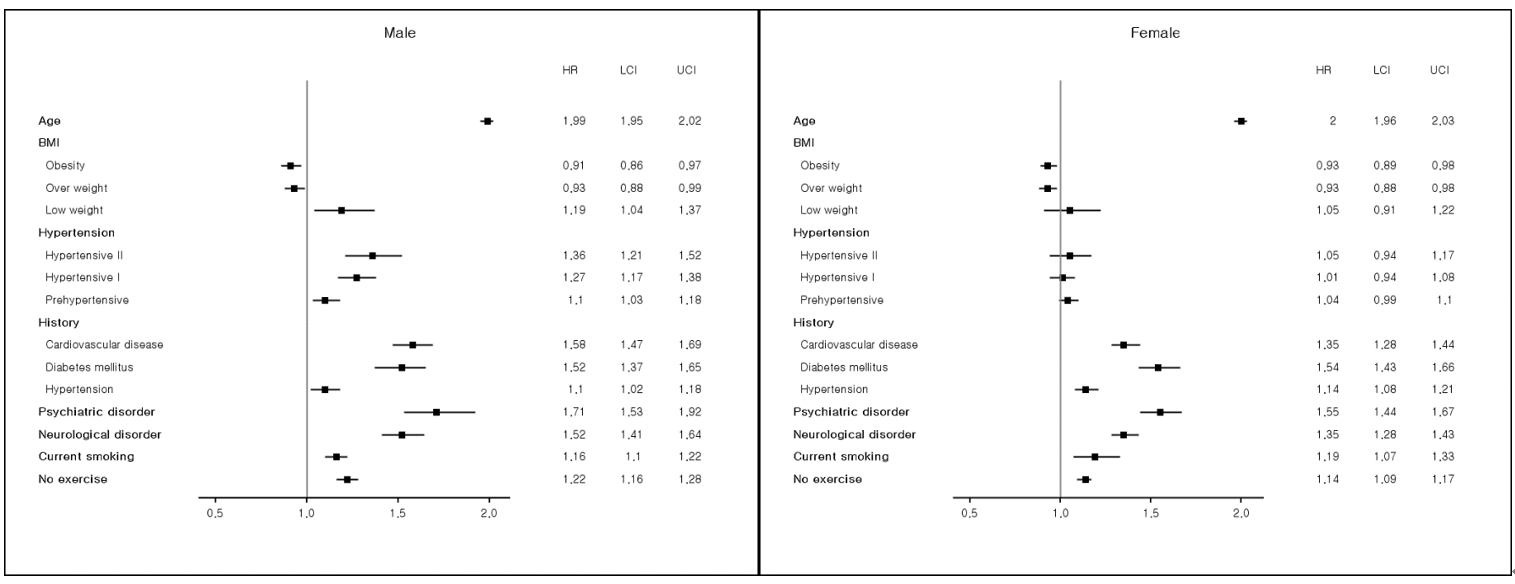

Supplement: S4 Fig — (TIF) [file pone.0211957.s008.tif]
